# Virtual Spectral Selectivity in a Modulated Thermal Infrared Emitter with Lock-In Detection

**DOI:** 10.3390/s22145451

**Published:** 2022-07-21

**Authors:** David Santalices, Juan Meléndez, Susana Briz

**Affiliations:** LIR—Infrared Laboratory, Department of Physics, Universidad Carlos III de Madrid, 28911 Leganés, Spain; juan.melendez@uc3m.es (J.M.); sbriz@fis.uc3m.es (S.B.)

**Keywords:** thermal emitter, blackbody, lock-in amplifier, Fourier series, optical gas sensor, gas detection, virtual emitter, methane, CO_2_, spectral selectivity

## Abstract

The need for affordable low-power devices has led MEMS-based thermal emitters to become an interesting option for optical gas sensors. Since these emitters have a low thermal mass, they can be easily modulated and combined with a lock-in amplifier for detection. In this paper, we show that the signal measured by a lock-in amplifier from a thermal emitter that varies its temperature periodically can have different spectral profiles, depending on the reference signal used. These virtual emitters appear because the Fourier series expansion of the emitted radiance, as a function of time, has different coefficients for each wavelength, and this spectral signature, which is different for each harmonic, can be retrieved using a reference signal that corresponds to its frequency. In this study, the effect is first proved theoretically and then is measured experimentally. For this purpose, we performed measurements with an IR camera provided with six different spectral filters of a modulated emitter, in combination with lock-in amplification via software. Finally, we show a potential application of this effect using multiple virtual emitters to gain spectral selectivity and distinguish between two gases, CO${}_{2}$ and CH${}_{4}$.

## 1. Introduction

Optical gas sensors are devices designed for in situ and real-time detection and measurement of different gases. Their operation is based on the Beer–Lambert law, which relates the attenuation of light that travels through a gaseous medium to the absorption coefficient of the gas, the length of the optical path, and the gas concentration. The absorption coefficient depends on the wavelength and is a characteristic of each gas, which means that each gas absorbs at specific spectral lines or bands.

Nearly all gases (except monatomic or diatomic homonuclear gases) have absorption bands in the infrared (IR) region, and most optical gas sensors operate in that spectral band. Therefore, we focus henceforth on IR gas sensing. Typically, IR gas sensors comprise an IR emitter, a wavelength-selection device, and an IR detector [1]. They operate by comparing signals in two channels: a reference channel, unaffected by gas absorption, and a measurement channel whose signal depends in a known way on the concentration of the target gas. Therefore, an optical gas sensor must have some degree of spectral selectivity to operate properly.

The most simple way to achieve spectral selectivity is to use a broadband light source with different optical interference filters tailored to the spectral features of the gases to be monitored. There are, however, many alternatives: laser sources are very common, as well as more complex systems, such as those based on diffraction gratings or interferometry; devices specifically designed for gas sensing, such as rotating gas-filter wheels, have also been used because of their excellent specificity [2,3].

As the scope of environmental regulations broadens worldwide [4], the need for simple and cheap gas sensors increases. This study focuses on systems that use an inexpensive source developed in recent years: thermal emitters based on micro-electro-mechanical systems (MEMS). These devices present some advantages over conventional thermal emitters. Their compact size, low power consumption, and low cost make them good candidates for developing optical gas sensors. Because of these features there is an increasing number of optical gas sensors using this technology [5,6,7,8,9,10,11].

An especially interesting feature of MEMS-based thermal emitters is that they can be used as modulated IR emitters because of their low thermal mass. This makes it possible to use lock-in detection to improve the signal-to-noise ratio [1,5,6,7,8]. A lock-in amplifier extracts the signal at a certain frequency from the total signal received by the detector, multiplying it by a reference signal at that frequency and performing the integral of the product over a period of time. Since signals with a frequency different from the reference integrate to zero after multiplication, this technique allows retrieval of small signals, even if they are embedded in high-bandwidth noise.

The necessary modulation can be achieved in different ways. In some applications, it is provided by the natural pulsating characteristics of the source [12,13], but the most usual method in optical sensing is to use an external chopper that turns on/off the light of the emitter. When using MEMS, however, their small thermal inertia makes it possible to achieve modulation by using a modulating power supply that leads to a periodic change in the emitter’s temperature. This implies a change in the integrated radiance, but, according to Planck’s law, also in the spectral signature, in contrast to the usual on/off modulation provided by a chopper.

This modulation of the spectral signature that can be observed in MEMS has been generally overlooked. It follows, as will be explained in this paper, that in this case, lock-in detection can lead to a significant change in the effective temperature and spectral signature of a thermal emitter. More specifically, a thermal source whose power supply is modulated at a certain frequency behaves, when submitted to lock-in detection, as a series of “virtual” light sources with different spectral profiles (and, therefore, different effective temperatures). These light sources correspond to the successive harmonics of the emitted radiance, and the signal of each one can be measured independently using lock-in detection, where the reference signal is at the same frequency as the harmonic to be measured.

This study aims to model this effect theoretically, verify it experimentally, and use it in a specific gas-sensing application. Section 2 explains the basic physics involved, builds a model of the source modulation and the operation of the lock-in detector, and applies it to a theoretical example to show the appearance of the virtual sources and their different spectral profiles. Section 3 verifies the effect in an experimental setting using a mid-IR camera and an MEMS-based thermal emitter. Section 4 demonstrates that it is possible to discriminate between CH${}_{4}$ and CO${}_{2}$ with a single-broadband detector and a single thermal source using power-supply modulation and lock-in detection. Finally, Section 5 reviews and critically summarizes the results obtained.

## 2. Theoretical Modelling

The spectral radiance [W/m${}^{2}\;\mu$m·sr] emitted by a thermal source with emissivity ϵ at temperature T [K] can be written as:

(1) $$L_{0}\left(\lambda ,T\right)=\epsilon \left(\lambda \right)L_{BB}\left(\lambda ,T\right)$$

The blackbody emission is given by Planck’s law:

(2) $$L_{BB}\left(\lambda ,T\right)=\frac{2hc^{2}}{\lambda^{5}}\frac{1}{e^{\frac{hc}{\lambda k_{B}T}}-1}$$

where *h* is Planck’s constant, *c* is the speed of light and $k_{B}$ is Boltzmann’s constant. The possible dependence of emissivity ϵ on temperature and emission angle has been omitted for simplicity. The blackbody radiance of Equation (2) peaks at a wavelength given by Wien’s displacement law:

(3) $$\lambda^{peak}=\frac{b}{T}$$

with b≈2898μm·K.

In the following subsections (Section 2.1 and Section 2.2), the spectral effect of implementing lock-in amplification with a temperature-varying emitter is modelled. In addition, we verify this effect with a theoretical example (Section 2.3).

### 2.1. IR Source Modelling

It will be assumed that the temperature of the emitter is modulated, so that we can write T=T(t) and $L_{0}=L_{0}\left(\lambda ,t\right)$. If the modulation frequency is *f* and the period $P\equiv f^{-1}$, $L_{0}$ will be a periodic function of *t* that can be Fourier-expanded:

(4) $$L_{0}\left(\lambda ,t\right)=a_{0}\left(\lambda \right)+\sum_{n=1}^{\infty }\left[a_{n}\left(\lambda \right)cos\left(2\pi nft\right)+b_{n}\left(\lambda \right)sin\left(2\pi nft\right)\right]$$

Since $L_{BB}$ is not linear in *T*, the Fourier coefficients ($a_{0}$, $a_{n}$, $b_{n}$) will be different from those of the expansion of T(t) and, since the periodic variation of $L_{BB}$, both in amplitude and in shape, is different for each wavelength, they will depend on λ, as shown by the notation used in Equation (4). Their explicit expressions are:

(5) $$a_{0}\left(\lambda \right)=\frac{1}{P}\int_{P}L_{0}\left(\lambda ,t\right)dt$$

(6) $$a_{n}\left(\lambda \right)=\frac{2}{P}\int_{P}L_{0}\left(\lambda ,t\right)\cdot cos\left(2\pi nft\right)dt$$

(7) $$b_{n}\left(\lambda \right)=\frac{2}{P}\int_{P}L_{0}\left(\lambda ,t\right)\cdot sin\left(2\pi nft\right)dt$$

When the radiance travels through a medium with transmittance τ(λ) to a detector with responsivity r(λ), a signal $s_{D}$ is generated:

(8) $$S_{D}\left(t\right)=\int_{0}^{\infty }\tau \left(\lambda \right)r\left(\lambda \right)L_{0}\left(\lambda ,t\right)d\lambda$$

Therefore, the signal $S_{D}$ will contain the same frequencies as $L_{0}$. In the next subsection, how a lock-in amplifier measures the different harmonics of the source ($a_{n}$, $b_{n}$) is described.

### 2.2. Modelling of the Lock-In Amplifier

A technique commonly used to improve the signal-to-noise ratio is lock-in detection. A lock-in amplifier multiplies an input signal $S_{D}\left(t\right)$ by a periodic reference signal $S_{R}\left(t\right)$ and integrates the result over a specified time. Its operation principle relies on the orthogonality of the sinusoidal functions. This means that, for sufficiently long integration times, signals with the same frequency and phase as the reference signal give a continuous output, while signals whose frequency spectrum does not overlap with the reference cancel out on integration.

The output measured by the lock-in amplifier can be written as [14]:

(9) $$S_{L}=\frac{2}{P}\int_{P}S_{R}\left(t\right)S_{D}\left(t\right)dt$$

(the 2/P factor is the normalization constant). For simplicity, here, a noiseless input signal with a frequency *f* is assumed. This lock-in output can be made more explicit using the Fourier expansions of $S_{R}$ and $S_{D}$. First, for the reference signal, we write:

(10) $$S_{R}\left(t\right)=A_{0}+\sum_{n=1}^{\infty }\left[A_{n}cos\left(2\pi nft\right)+B_{n}sin\left(2\pi nft\right)\right]$$

Then, using the expression (8) for $S_{D}$ and the Fourier expansion (4) for $L_{0}$, it is found that

(11) $$S_{L}=2A_{0}\int_{0}^{\infty }\tau \left(\lambda \right)r\left(\lambda \right)a_{0}\left(\lambda \right)d\lambda +\sum_{n=1}^{\infty }\left[A_{n}\int_{0}^{\infty }\tau \left(\lambda \right)r\left(\lambda \right)a_{n}\left(\lambda \right)d\lambda +B_{n}\int_{0}^{\infty }\tau \left(\lambda \right)r\left(\lambda \right)b_{n}\left(\lambda \right)d\lambda \right]$$

In the simplest case, the reference signal is just a sinusoid of unit amplitude, i.e., $S_{R}\left(t\right)=\cos\left(2\pi ft\right)$ and then $S_{L}=\int_{0}^{\infty }\tau \left(\lambda \right)r\left(\lambda \right)a_{1}\left(\lambda \right)d\lambda$, which means that the lock-in signal is the response to the first harmonic component $a_{1}\left(\lambda \right)$ rather than to the full radiance $L_{0}$. In practical measurements, since it cannot be assured that $S_{R}$ is in phase with $L_{0}$, the most typical lock-in configuration uses a sine and a cosine wave as independent references and the two responses are added up in quadrature.

In this study, however, the general expression of the lock-in signal, Equation (11), will be used. In this expression, the first term is due to the average radiance, i.e., the DC term $a_{0}\left(\lambda \right)$, and each term in the summation is the contribution of the successive amplitudes of the harmonics of the modulated radiance, $a_{n}\left(\lambda \right)$ and $b_{n}\left(\lambda \right)$, weighted by the corresponding amplitudes of the reference signal, $A_{n}$ and $B_{n}$. Therefore the total signal measured by a lock-in amplifier when using a modulated broadband source can be viewed as the sum of responses to a series of sources with radiances equal to $2A_{0}a_{0}\left(\lambda \right)$ and $\left\{A_{n}a_{n}\left(\lambda \right),\;\;B_{n}b_{n}\left(\lambda \right)|n\in N\right\}$. Thus, each harmonic component works as a “virtual” source. The spectral radiance of the first source is, not surprisingly, the time average of the spectral radiance of the source. However, the rest of the harmonics have different spectral profiles. Since the reference signal can be chosen at will, it is possible to make any of these terms zero and isolate the contribution of each “virtual” source. This effect is explained with a specific example in the next section.

### 2.3. Spectral Profiles of Harmonic Amplitudes: Theoretical Example

As previously explained, each Fourier component of the radiance emitted by a modulated thermal source will have a different spectral signature. To illustrate this, we have calculated them for a blackbody thermal emitter whose temperature has a sinusoidal variation, as shown in the following equation:

(12) T=725+75cos(2πft)[K]

**Figure 1:**
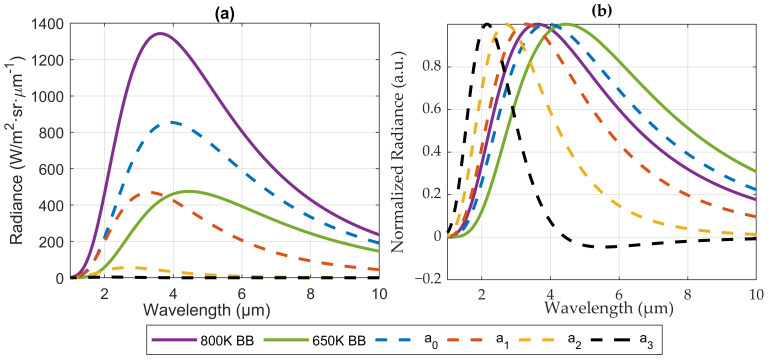
(**a**) Spectra of the first Fourier amplitudes of the radiance of a blackbody source whose temperature oscillates sinusoidally between T=650 K and T=800 K, compared to the blackbody spectra. (**b**) The same spectra of (**a**), normalized.

Numerical integration over a period of time of Equations (5)–(7), leads to the spectra associated with each harmonic ($a_{0}\left(\lambda \right)$, $a_{n}\left(\lambda \right)$, $b_{n}\left(\lambda \right)$). The result is shown in Figure 1a, where the spectra of the blackbodies at the extreme temperatures (650 K and 800 K), are compared to those of the first Fourier amplitudes of the modulated radiance. For a sinusoidal modulation of *T* as in Equation (12), high-order harmonics are not zero due to the non-linear relation between temperature and radiance but, as expected, their intensity decreases for increasing order (only the first cosine terms $a_{1},\;a_{2},\;a_{3}$ have been represented; for a cosine modulation as in (12), the sine terms $b_{n}$ are zero).

For an instrument measuring in continuous mode (i.e., simply integrating the signal over time), the effective spectral signature of the source would be $a_{0}\left(\lambda \right)$, intermediate between those of the extreme temperatures of the source. However, for an instrument that measures in a lock-in mode, the spectra of the harmonics are shifted to shorter wavelengths: the peak wavelength of the blackbody spectrum at T=800 K is $\lambda_{T1}^{peak}=\;3.62\;\mu$m, whereas for the first harmonic $\lambda_{a1}^{peak}=\;3.29\;\mu$m. This effect is larger for higher harmonics: $\lambda_{a2}^{peak}=\;2.68\;\mu$m and $\lambda_{a3}^{peak}=\;2.14\;\mu$m, as can be best appreciated in Figure 1b, where the spectra of blackbody and harmonics have been normalized to make the comparison easier.

This result means that, for each harmonic, the lock-in “sees” a source with a different spectral signature and, consequently, a source with a different effective temperature. For instance, to obtain peaks of emission at the same wavelengths of $a_{1}\left(\lambda \right)$, $a_{2}\left(\lambda \right)$, and $a_{3}\left(\lambda \right)$, blackbodies at temperatures of 881 K, 1081 K and 1354 K, respectively, should be used. Therefore, using lock-in amplification to detect radiation from a thermal source with modulated temperature makes it possible to confer some degree of spectral selectivity to an ordinary blackbody source. It is important to bear in mind that this effect is a consequence of the non-linear dependence of Planck’s law (Equation (2)) on temperature, and therefore only appears if modulation of the radiation is achieved by changing the source temperature, rather than using an external modulating device, such as an optical chopper.

## 3. Experimental Verification

An experimental test was performed to verify the theoretical results of Section 2; in particular, we sought to check experimentally the spectral dependence of the harmonics of the radiance emitted by the thermal source, as depicted in Figure 1.

For this purpose, we required a detector system to measure the IR radiation emitted by the source with a certain spectral resolution and a methodology based on the lock-in technique that would allow us to spectrally analyze the harmonics of the signal as measured by the system.

### 3.1. Experimental Setup

The basic experimental setup was made up of a thermal emitter and a detector system, composed of an IR camera equipped with a series of interference filters that give it the ability to perform the required spectral analysis.

The infrared emission of a time-varying thermal source was measured by the IR camera in six spectral regions defined by the optical filters. The thermal emitter used was the JSIR 350-4 [15], an off-the-shelf MEMS-based emitter with a blackbody-like pattern emission. It was modulated at a frequency of 10 Hz and varied its temperature between approximately 650 and 800 K.

The detection was performed using a Thermosensorik SME 640 camera with an InSb Stirling-cooled FPA detector. The camera operates in the mid-IR, with a spectral range from 1.5 μm to 5 μm. Combining a short integration time (ranging from 0.03 to 0.05 ms) with a 32 × 32 pixel subarray, it is able to reach frame rates of ∼1000 frames per second.

**Figure 2:**
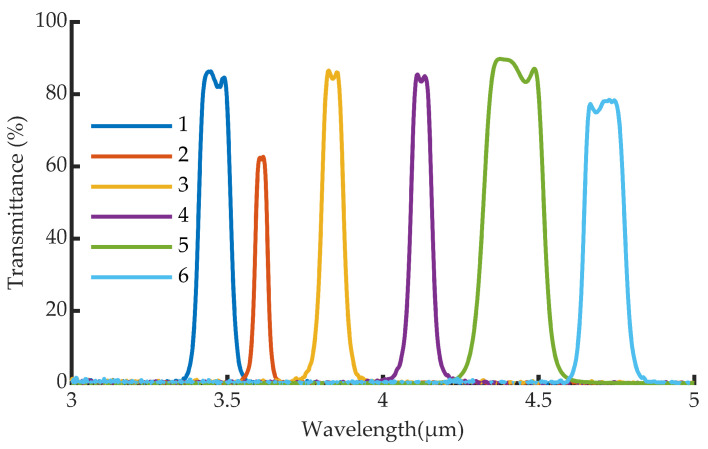
Spectral transmittance of the six interference filters used in the infrared camera. Each color represents a different optical filter.

The camera has a wheel with six selectable interference filters that provides spectral selectivity. The spectral transmittance of the filters is shown in Figure 2.

### 3.2. Methodology

The procedure followed in the experiment consisted of calibration of the IR camera, measurement and processing.

#### 3.2.1. Calibration

In order to measure radiance values at each spectral channel defined by the filters, a specific radiometric calibration must be performed. The objective is to obtain, from the digital number (DN) provided by the IR camera, the spectral radiance that reaches the system.

An extended blackbody is measured at different temperatures ($T_{j}$) by the camera with each filter. Integration of the product of the spectral radiance of the blackbody by the spectral transmittance of the filter provides the incoming radiance after passing through each filter $L_{i}^{in}\left(T_{j}\right)$ [W/m${}^{2}\cdot$sr]. For each filter i, a linear least-squares fitting provides a Gain${}_{i}$ and an Offset${}_{i}$ that relate the measured digital number DN${}_{i}$ to the incoming radiance $L_{i}^{in}$.

#### 3.2.2. Measurements

Once the camera is calibrated, the variable IR emitter replaces the blackbody. Then, measured values of $L_{i}^{in}\left(t\right)$ are divided by the integral of the filter transmittance to obtain values of effective experimental spectral radiance, $L_{i}^{exp}$ [W/m${}^{2}\mu$m·sr]:

(13) $$L_{i}^{exp}\left(t\right)=\frac{L_{i}^{in}\left(t\right)}{\int_{0}^{\infty }\tau_{i}\left(\lambda \right)d\lambda }$$

where $\lambda_{i}$ is the central wavelength of the i-th filter. If the filter is sufficiently narrow, this effective spectral radiance will be very similar to the real one, $L_{i}^{exp}\left(t\right)∼L\left(\lambda_{i},t\right)$

#### 3.2.3. Post-Processing

**Figure 3:**
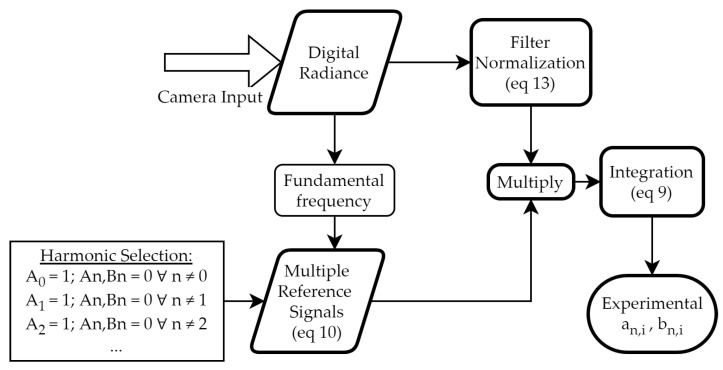
Flowchart of the post-processed lock-in amplification.

The experimental effective spectral radiances must now be post-processed to obtain the different harmonics ($a_{n,i}^{exp}\left(\lambda_{i}\right)$, $b_{n,i}^{exp}\left(\lambda_{i}\right)$). Figure 3 summarizes the procedure.

First, the fundamental frequency of the signal (in frames) is calculated using a fast Fourier transform (FFT). Then, for each harmonic to be measured ($a_{n}$, $b_{n}$), it is necessary to create a reference signal (Equation (10)) whose coefficients ($A_{n}$, $B_{n}$) are zero, except the coefficient of the harmonic to be measured, which is set to one (please note that in this study the technique is used to select the harmonics, not to amplify them, and the process is numeric, not electronic). By substituting in Equation (11), we obtain that the experimental harmonic measured by the lock-in, $a_{n,i}^{exp}\left(\lambda_{i}\right)$, is:

(14) $$a_{n,i}^{exp}\left(\lambda_{i}\right)=\int_{P}L_{i}^{exp}\left(t\right)\cos\left(2\pi nft\right)dt$$

The same processing applies for the coefficients $b_{n,i}^{exp}$. To avoid phase shift issues, the Cartesian coefficients ($a_{n}$, $b_{n}$) have been transformed into the polar amplitude, $c_{n}=\sqrt{a_{n}^{2}+b_{n}^{2}}$.

### 3.3. Results

**Figure 4:**
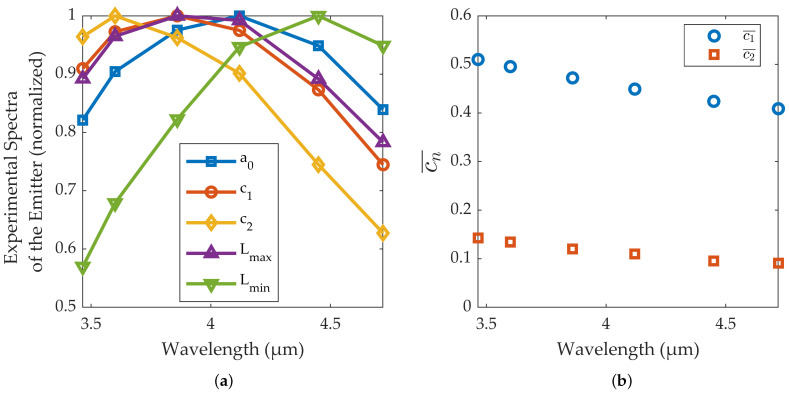
(**a**) Continuous part of the signal ($a_{0}$), first and second harmonics ($c_{1}$ and $c_{2}$), and maximum and minimum radiance of the emitter during each cycle ($L_{max}$ and $L_{min}$), for each of the six filters used. (**b**) Amplitudes of the first two harmonics for each spectral band normalized to the DC signal.

The continuous part of the signal ($a_{0}$), the first and second harmonics ($c_{1}$ and $c_{2}$), and the maximum and minimum radiance of the emitter during each cycle ($L_{max}$ and $L_{min}$) for each filter are shown in Figure 4a. The different harmonics and radiances have been normalized to the unit to make their comparison easier.

The results in this figure must be compared to those obtained for the theoretical example, shown in Figure 1b. In both graphs, $a_{o}$ (blue curve) shows a peak wavelength intermediate between those of the extreme temperatures of the source. However, the harmonics measured by the lock-in ($c_{1}$ and $c_{2}$ in Figure 4a) have a spectral signature shifted towards shorter wavelengths, as $a_{1}$ and $a_{2}$ in the theoretical example of Figure 1b. Thus, it is demonstrated that the harmonics of the emitter show a peak wavelength shorter even than the emitter’s peak wavelength when it reaches maximum temperature during its cycle. This result shows that implementing lock-in amplification with this thermal emitter is equivalent to effectively increasing its temperature, in terms of peak wavelength.

Although Figure 1b and Figure 4a show the same overall spectral pattern, there are differences in the values of the peak wavelengths. One reason is the spectral dependence of the emissivity of the source (further information in [15]) which affects the $L_{min}$, $L_{max}$, $a_{0}$, $c_{1}$ and $c_{2}$ curves in Figure 4. In addition, the thermal inertia of the emitter and the non-linear relationship between the power supply and the emitter temperature also cause a non-sinusoidal variation of the source temperature. Although this effect does not change the $L_{min}$ and $L_{max}$ curves, it does affect the $a_{0}$, $c_{1}$ and $c_{2}$ curves.

Figure 4b shows the amplitudes of the first two harmonics for each spectral band normalized to the DC signal. We will denote as $\bar{c_{n}}$ these normalized amplitudes:

(15) $$\bar{c_{n}}=\frac{c_{n}}{a_{0}}$$

Since the spectral profiles of the DC signal and the harmonics are different, these ratios change throughout the spectral band of the camera. Therefore, the measured values of $\bar{c_{n}}$ will be different according to the spectral composition of the incoming light. In our case, Figure 4b shows that light near the 3 μm region will give larger $\bar{c_{1}}$ and $\bar{c_{2}}$ values than light near the 5 μm region. This means that it is possible to have some spectral sensitivity in a single broadband camera, e.g., 3 to 5 μm, using a broadband infrared source.

## 4. Application to Gas Detection

A potential application of the effect just described is the use of multiple virtual sources to differentiate between different gases in a gas cell with a single band camera, simply by modulating the thermal emitter.

The principle on which optical gas sensors are based is the Beer–Lambert law, that describes the attenuation of a ray as it passes through a medium:

(16) $$L\left(\lambda \right)=L_{0}\left(\lambda \right)e^{-\alpha (\lambda )LC}$$

where L is the measured spectral radiance, $L_{0}$ the spectral radiance emitted by the IR source, *L* is the optical path travelled within the gas, α the absorption coefficient of the selected gas, and C its concentration. A useful way to express the Beer–Lambert law is with the column density, which is the concentration times optical path, i.e., the LC factor of the expression (16). Since α is non-zero only at specific wavelengths for each gas, a proper selection of the incoming radiance is essential to define a signal and a reference channel.

As explained in the previous sections, modulation of a thermal emitter (varying its temperature) in combination with lock-in detection creates several virtual sources with different spectral signatures. These virtual emitters provide some degree of spectral selectivity that makes it possible to distinguish two gases using a single-band detector. In this section, we apply this idea to CH${}_{4}$ and CO${}_{2}$.

**Figure 5:**
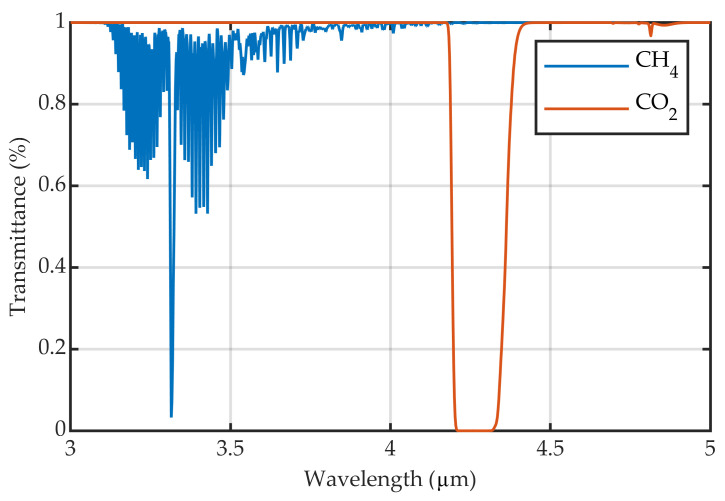
Transmission spectra of pure CO${}_{2}$ and a mixture of CH${}_{4}$ and N${}_{2}$; the mole fractions of the gas mixture are 0.15 and 0.85, respectively. Both gases at ambient temperature and pressure for an optical path of 20 cm. The spectra have been obtained using the HITRAN database [16].

In order to demonstrate the effect, a gas cell was observed with the IR camera previously described against the thermal emitter as the background. A 3–5 μm filter was used in the camera. The gas cell was filled initially with air and then was progressively filled with CO${}_{2}$ and, in a second experiment, with a gas mixture of CH${}_{4}$ and N${}_{2}$. All gases were at ambient temperature and pressure. These gases were chosen because both have absorption bands in the spectral range of the detector (3–5 μm). Nevertheless, within this region, CH${}_{4}$ absorption is located between 3.2 and 3.6 μm, while CO${}_{2}$ absorption is more localized between 4.2 and 4.3 μm (Figure 5).

**Figure 6:**
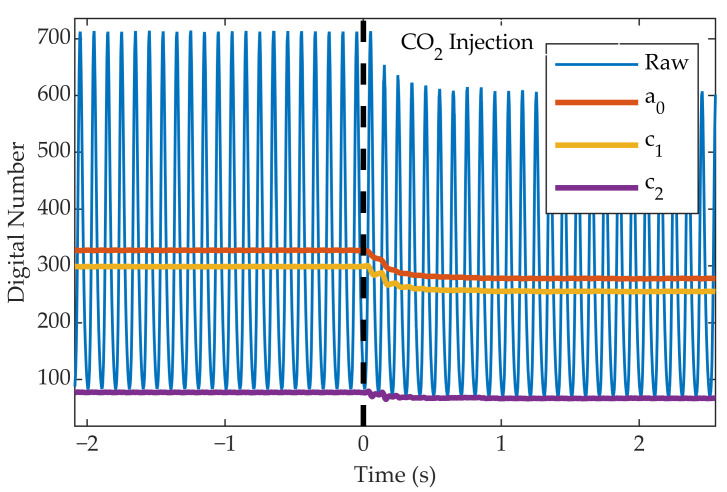
Signal measured by the camera when one of the gases is injected into the cell (in this case CO${}_{2}$).

When a gas is injected into the gas cell, a decrease is observed in the value of the raw signal and the post-processed harmonics. Figure 6 shows this effect for the CO${}_{2}$ injection. Therefore, using the signal of a single harmonic, it is possible to calculate the integrated absorption of the medium for its spectral profile and, if a single known gas is present in the gas cell, to obtain its concentration (using the Beer–Lambert law (Equation (16)). However, if we do not assume which gas is present in the gas cell, in principle we cannot determine if the signal decreases because of the presence of CO${}_{2}$ or CH${}_{4}$. Nevertheless, the combination of multiple harmonics will enable us to discriminate whether this absorption is due to CO${}_{2}$ or CH${}_{4}$.

**Figure 7:**
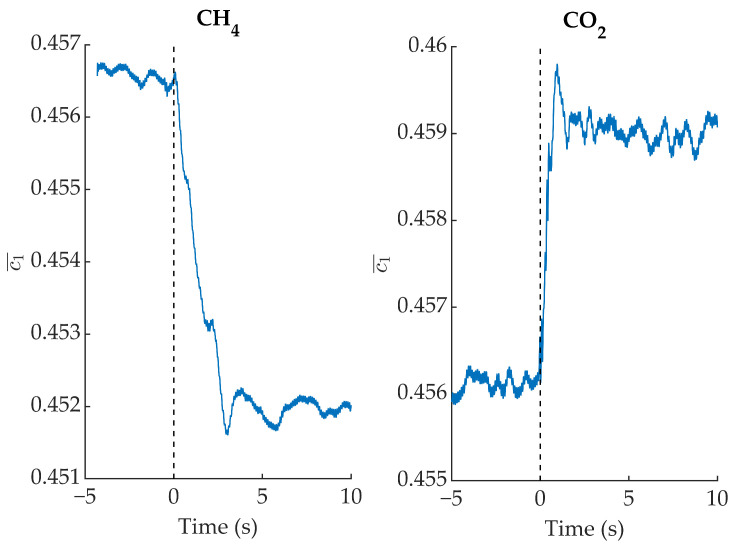
$\bar{c_{1}}$ evolution when CH${}_{4}$ and CO${}_{2}$ are introduced into the gas cell.

For a gas cell filled with air, $\bar{c_{1}}$ will assume a value which is approximately the average in the spectral band of the camera (3μm to 5μm). However, when it is filled with CH${}_{4}$, the contribution of short wavelengths decreases (due to absorption in the range between ≈3.2μm and ≈4.0μm). These wavelengths have large $\bar{c_{1}}$ values (see Figure 4b), and therefore the measured $\bar{c_{1}}$ value will decrease. On the other hand, when the gas cell is filled with CO${}_{2}$, it is the contribution of long wavelengths (between ≈4.2μm and ≈4.4μm), with small $\bar{c_{1}}$ values, that decreases, and thus the measured $\bar{c_{1}}$ value should increase. This effect is shown in Figure 7, where $\bar{c_{1}}$ is plotted when CO${}_{2}$ and CH${}_{4}$ are injected alternatively into the gas cell.

Since both gases were not measured simultaneously, it can be argued that a specific combination of CH${}_{4}$ and CO${}_{2}$ concentrations could cause the $\bar{c_{1}}$ not to change. However, even if $\bar{c_{1}}$ remains unchanged, a decrease in the signal received from $a_{0}$ and $c_{1}$ would be seen. With a direct model of these signals for different concentrations of CH${}_{4}$ and CO${}_{2}$ one can define and solve the inverse problem, obtaining the density column of both gases from the $a_{0}$ and $c_{1}$ values.

Because of the low modulation frequency, it is necessary to have large integration times in the lock-in to reduce noise from the system fluctuations. In this study, the integration time implemented is 0.4 s. However, this integration time could be longer in less stable systems (e.g., uncooled detectors).

## 5. Summary, Conclusions and Perspectives

When a thermal emitter varies its temperature periodically, it changes its spectral signature both in shape and intensity. This is because temperature and wavelength are non-separable variables in Planck’s law (Equation (2)). Therefore, the Fourier series expansion of the emitted radiance (as a function of time) will have different coefficients for each wavelength. This spectral dependence means that each harmonic has a different emission spectrum and can be conceived of as an independent “virtual” source.

Implementing lock-in amplification with one of these modulated thermal emitters will result in a measured spectral signature that depends on the lock-in reference signal and is different from the time average of the radiance received. Therefore, it is necessary to correctly model this effect in sensors that use a combination of thermal emitters with lock-in amplifiers (e.g., optical gas sensors). Although these sensors typically use an empirical calibration, this effect may be relevant during the design stage.

As shown in this study, the spectral profile of the successive harmonics is progressively shifted to shorter wavelengths. Their amplitude, however, is limited by the extreme values reached by the emission spectra during the modulation ($L_{max}$ and $L_{min}$), which decrease most rapidly for the shortest wavelengths, and also decay as the order *n* of the harmonic increases. Therefore, although the number of virtual sources is, in theory, infinite, their usefulness is generally limited to the first few harmonics. This range of application can be extended, however, if the interval between the maximum and minimum temperatures of the source is increased.

This, however, is enough to solve detection problems that otherwise could not be addressed without specific wavelength-selection devices (such as interference filters) or spectrally selective sources (such as infrared lasers). In this study, it has been demonstrated that a very simple system, cheap and with no moving parts, using a thermal emitter and a broadband mid-IR detector, is able to distinguish between CH${}_{4}$ and CO${}_{2}$ using only a combination of the amplitudes $a_{0}$ and $c_{1}$, readily measurable by lock-in processing. Even quantification is, in principle, possible by modelling the direct problem that relates absorption to column density, and then solving the inverse problem. This possibility is particularly appealing since CH${}_{4}$ and CO${}_{2}$ are the two main greenhouse gases and there is a pressing need for cheap sensors for both of them [17].

## Data Availability

Not applicable.
